# Determinants of practice for decision coaching in Germany: a qualitative exploration of decision coaches’ perspectives

**DOI:** 10.1186/s12913-025-13752-z

**Published:** 2025-12-01

**Authors:** Lia Schilling, Birte Berger-Höger

**Affiliations:** Institute for Public Health and Nursing Research, University of Bremen, Bremen, Germany

**Keywords:** Decision coaching, Decision support interventions, Patient participation, Informed choice, Barriers and facilitators

## Abstract

**Background:**

Patients often want to be involved in decisions about their health. However, to actively participate in the decision-making process and make informed choices, they often require support. Decision coaching can be offered as a non-directive form of decision support provided by trained health care professionals. So far, decision coaching has only been tested in pilot projects in Germany. To investigate the determinants of practice that influence implementation, decision coaches were interviewed in a qualitative study about their practical experiences with decision coaching and regarding facilitators and barriers in its implementation.

**Methods:**

Guided expert interviews were conducted from June 2023 to November 2023 with decision coaches from model projects in Germany. The decision coaches were asked about their tasks, work processes, framework conditions, understanding of their role, self-perception, the decision support tools used and barriers and facilitators for the implementation of decision coaching. Sociodemographic data were collected anonymously. The verbatim transcribed interviews were analysed using content analysis according to Kuckartz with MAXQDA. Barriers and facilitators of the implementation of decision coaching were analysed using the Consolidated Framework for Implementation Research (CFIR 2.0) framework.

**Results:**

Seven guided interviews were conducted with experienced female decision coaches (Ø 56 years) from pilot projects in oncology and neurology. While some elements were sustained in practice, only one project continued long-term. Overall, the decision coaches strongly identified with their role and patient acceptance was high. Thematic analysis revealed seven main categories with 32 subcategories. Crucial factors influencing the implementation included structured training, flexible delivery formats, institutional support, dedicated space and funding. Although decision coaching was perceived to enhance patient preparedness and physician-patient communication, structural challenges remain.

**Conclusion:**

The coaches perceived their role as supportive in patients’ decision-making. However, the effectiveness and implementation of decision coaching are strongly influenced by several contextual factors that could guide the refinement of interventions and the selection of implementation strategies.

**Clinical trial number:**

Not applicable.

**Supplementary Information:**

The online version contains supplementary material available at 10.1186/s12913-025-13752-z.

## Background

Most patients want to be involved in decisions about their health [1, 2]. However, making informed health decisions has become increasingly challenging due to the growing complexity of medical options for patients [3, 4]. In addition, many patients experience a different role in decision-making than they preferred [5, 6]. Patients often face significant barriers, including a lack of understanding about the risks and benefits of various treatments and uncertainty about how their personal values align with the available options. The Ottawa Decision Support Framework (ODSF) identifies these challenges, with common decisional needs reported by patients including feeling uninformed, unsupported and uncertain in their decision-making process [7, 8]. The ODSF is an evidence-based framework that has been developed to assist individuals in navigating health and social decisions. The framework proposes that optimal decisions are achieved when the individual decisional needs are identified and appropriate decision support is provided [8].

To address these challenges, shared decision making (SDM) and decision support interventions like evidence-based decision aids, decision guidance and decision coaching are used to support patients in navigating complex healthcare choices [9–12]. These approaches aim to improve patients’ knowledge of available options, encourage reflection on personal preferences and values and prepare them to participate in medical consultations [9].

SDM is a collaborative process between patients and their healthcare team to ensure informed decisions. It emphasises the open exchange of relevant information, with healthcare providers presenting all medical options clearly and neutrally, while patients evaluate them based on their individual needs and preferences [13, 14]. Decision aids offer evidence-based information in a clear, balanced way for patients facing health decisions. They outline the decision to be made, provide information on the condition, present options with associated risks and benefits, address uncertainties and include exercises to clarify patients’ values and preferences [10, 15]. A decision guide is a structured tool that supports patients in making informed health decisions independently. It can include various elements, such as step-by-step approaches, worksheets to clarify personal values, questions to ask healthcare providers or automated summaries of patients’ priorities and needs [10, 16, 17].

Decision coaching is a non-directive support for patients provided by healthcare professionals, who have been trained as decision coaches to help patients actively participate in healthcare decisions [16]. Decision Coaching is designed for patients who are facing a decision or wish to review a decision they have already made [17, 18]. Decision coaches assist patients in identifying their needs and preferences, understanding evidence-based information about all available options, including the benefits and risks and clarifying their personal values regarding the outcomes of different options. They help patients communicate their preferences to others, such as family members or healthcare providers [9, 11, 16, 19]. The coaching process typically follows these steps: (a) assessing the patients’ decision-making needs; (b) providing information on their options including the benefits and risks (c) verifying their understanding; (d) clarifying their values associated with the attributes of the options and their attitude toward risks; (e) building their communication skills and accessing support; (f) screening for barriers to implementation and (g) facilitating progress in decision making [19]. This support aims to complements rather than replaces physician consultations by acting as a bridge between patients and healthcare providers [20]. Decision coaching can take place before or after a medical consultation and may consist of one or more sessions. The decision coaching sessions take place in a protected environment and can be conducted face-to-face, by phone, or via another communication channel. The decision coaches are not present at the consultations with the physicians; these are separate appointments. During the coaching decision coaches can provide patients with other evidence-based resources, such as evidence-based decision aids. Additionally, family members or other persons of trust may participate in the decision coaching at the patient’s request [16, 21, 22].

Systematic reviews of shared decision-making approaches emphasize the role of decision coaching in fostering patient independence and strengthening their ability to make informed choices [17, 19, 23]. There is limited evidence that decision coaching combined with evidence-based tools, such as patient decision aids, improve their knowledge without causing negative outcomes like decision regret or anxiety. For other outcomes, including decision-making preparation, confidence, feeling informed, having clear values or feeling supported, no definitive conclusions could be drawn [11]. Furthermore, the randomized controlled EDCP-BRCA trial reported that women were more satisfied with their role, had better knowledge and less decisional conflicts after receiving a structured nurse-led decision coaching [24].

Overall, research indicates that there are promising study results on the effectiveness of decision coaching. However, the determinants of practice of decision coaching have not yet been sufficiently investigated. The need for systematic studies to explore the factors that may either support or hinder the implementation of decision coaching was emphasised [20]. Most decision coaching interventions have taken place in pilot projects in several medical fields, including oncology, neurological conditions, cardiology and nephrology [25–27] and were used for various health decisions (e.g. treatment decisions, genetic testing or screening decisions) and have been conducted in multiple countries, most frequently in the USA, followed by Canada, Germany, the UK, Australia, Japan, and the Netherlands [11]. In Germany, decision coaching has not yet been integrated into routine care, but is also mainly carried out through pilot projects, for example in oncology and neurology [28, 29].

To improve research on decision coaching and promote its implementation in Germany, it is an opportunity to take a closer look at the determinants of practice s of decision coaching. Determinants of practice are factors that can act as barriers or facilitators of improvements in that practice [30]. The knowledge could inform further intervention development and implementation strategies as well. Against this background, the study seeks to answer what are the determinants that influence the practice of decision coaching in Germany from the perspective of decision coaches.

## Methods

We conducted a qualitative interview study to identify the determinants of practice of decision coaching in Germany. The data were collected through semi-structured, guided expert interviews with trained health care professionals who work as a decision coach or have been involved in a model project in the past in Germany. The results are reported in accordance with the Consolidated criteria for Reporting Qualitative research Checklist (COREQ) (Supplementary Material 1) [31].

### Conceptual model

To examine barriers and facilitators to the implementation of decision coaching in practice-based projects, the Consolidated Framework for Implementation Research (CFIR 2.0) was used [32]. CFIR 2.0 is a comprehensive implementation framework that offers a structured taxonomy of factors influencing implementation. It comprises five major domains encompassing a total of 40 constructs: the innovation itself, the inner setting (e.g., the specific organization where implementation occurs), the outer setting (e.g., broader systems such as hospital networks or regulatory environments), the individuals involved and the implementation process. CFIR 2.0 was well-suited for this analysis as it captures all socio-ecological levels relevant to implementation, including individual, organizational, community, system, and policy levels. By using CFIR 2.0 as a framework, we ensured a structured and comprehensive approach to understanding the determinants of practice of decision coaching.

### Sample and recruitment

We included individuals aged 18 or older who were employed as decision coaches in Germany in the last ten years, received a relevant training and worked in practice as decision coaches or were still actively engaged in this role at the time of the interview.

Participants were recruited through publicly available websites of research projects carried out in Germany in which decision coaches were employed. Additionally, authors of publicly accessible studies and publications from Germany on this topic were contacted. Contact was made via publicly available email addresses and phone numbers or through email addresses and phone numbers for which explicit consent for sharing/contact had been provided.

### Data collection and analysis

Data collection was conducted through semi-structured, guided expert interviews with current and former decision coaches from Germany. This form of interview was chosen to ensure maximum openness for participants while maintaining the ability to intervene in a structured manner during the interview. The interviews were conducted via Zoom and were led by two researchers (BBH and LS) or (IB and LS). The interviews were audio recorded with an external recorder and transcribed verbatim. Field notes were taken during the interviews by one researcher. The transcripts were not returned to the participants for checking. Sociodemographic data of the participants were collected anonymously via a questionnaire. These included information on age, gender, level of education, professional qualifications, current occupation and years of professional experience. Written informed consent was obtained from all participants prior to the interviews.

The interview guide (Supplementary Material 2) was divided into the following six main themes: General information about decision coaching, information on use, framework conditions, acceptance, barriers/ facilitators and institutionalization.

The qualitative content analysis was carried out by one researcher (LS) according to Kuckartz [33] with the software MAXQDA. A predefined category set based on the interview guide was applied to the interview data and supplemented by inductively derived subcategories during coding. The final category system (Supplementary Material 3) was applied to the entire material in a final analysis loop. The results of the analysis were then assigned to the CFIR 2.0 domains.

## Results

Seven female participants from the fields of oncology and neurology were interviewed from June 2023 to November 2023 (Table 1). The age range of the group was between 48 and 62 years. The participants were paediatric nurses, geriatric nurses, registered nurses and/or midwives. Six participants held additional qualifications, such as Breast Care Nurse and/or Study Nurse. The median time of professional experience was 35 years. No participants refused to participate or dropped out. All interviews were conducted digitally via the video conference system ZOOM, with an average duration of 38 min (range: 22–61 min).

**Table 1:** Baseline characteristic

Sex (*n* = 7)	Female	7
	Male	0
Age in years; median (range)		56 (48–62)
Education	Secondary School Certificate, Intermediate School Certificate (also transition to 11th grade of High School/Comprehensive School)	4
	University of Applied Sciences Entrance Qualification, High School Diploma, Vocational Diploma, Vocational Qualification (Vocational School)	2
	High School Diploma **and** Vocational Diploma (Vocational School)	1
Profession (Multiple answers)	Pediatric Nurse	5
	Geriatric Nurse / Registered Nurse	2
	Midwife	2
Years of professional experience in nursing median (range)		35 (20–48)

### Content analysis

The following seven main categories with 32 subcategories emerged from the content analysis: General Information on Decision Coaching, Framework Conditions, Support Needs of Decision Coaching, Utilization, Benefits of Decision Coaching, Acceptance, Sustainability and Maintenance. Given that determinants of practice may serve as barriers, facilitators, or both, we incorporated the identification of these factors within each thematic category to maintain a coherent analytical framework. Subsequently, we assigned the identified determinants of practice to the respective CFIR 2.0 domains. Since some determinants may relate to multiple domains, we decided to present our findings based on domains rather than categories. Each determinant was then classified as a facilitator or barrier to decision coaching. Specifically, under the *innovation itself domain*, we analysed the specific characteristics of each implemented decision coaching intervention. The *implementation process* domain captures which strategies or measures—such as the basic training of decision coaches—have been employed, as well as how they were implemented and examines their potential impact on the success of decision coaching implementation. We examined the *characteristics of individuals*, focusing on the decision coaching recipients (patients) and the deliverers (decision coaches), for example the role and self-image of decision coaches and their professional identity. The *inner setting* was explored by assessing the structural and organizational conditions influencing decision coaching, including available resources, institutional support and the perceived acceptance of decision coaching by patients, physicians, and other relevant interest-holders. Additionally, the *outer setting* was considered, as we evaluated the external factors that impact the implementation of decision coaching.

### Innovation itself domain

We summarised the specific characteristics of the individual decision coaching interventions within the innovation domain.

All participants took part in multi-day training sessions (1.5–3 days) held within the pilot projects, delivered by expert teams in decision-making and decision coaching. The training prepared the coaches for their role and included modules on communication skills, dealing with patients’ emotions, and the use of evidence-based health information and available decision-support tools. Participants’ prior experience was limited to their professional education and practical work as nurses or midwives; they had not previously received specific training in decision coaching. The decision coaching was firmly anchored in the clinical structures that patients go through during their decision-making process. The decision coaches reported that they were not present during the clinician consultations. Some of the decision coaches were also attending the tumour board meetings. In general, one decision coaching appointment was scheduled per patient. Follow-up appointments could be made if necessary. One decision coach reported that two appointments per patient were scheduled regularly. In some cases, the clinicians referred their patients to the decision coach. In other cases, patients could sign up themselves. The decision coaching took place on-site with the decision coach. During the SARS-COV2-pandemic, the decision coaching sessions were often offered online.

The timing of the decision coaching varied. In some cases, the decision coaching was scheduled either before or after the therapy consultation with the clinician - occurring immediately after diagnosis or prior to the treatment decision. In addition, not only newly diagnosed patients benefit from the decision coaching, but also those with an existing diagnosis who are, for example, re-evaluating their treatment plan. The decision coaches reported that the initial decision coaching session with each patient lasted between 1.5 and 2 h: *„The plan was one hour*,* but it always took me longer. Sometimes it took me two hours. Depending on what the women needed.*“ (A0104 pos. 5). The coaches reported that the topics of the coaching sessions included treatment options, side effects, prognosis, disease and treatment progression, psychological support, as well as private concerns and the patient’s environment.

Various structured tools, such as patient decision aids and prompt cards, were available to the decision coaches for use during the coaching sessions. Decision coaches reported that these tools were valuable tools in organising the dialogue and providing guidance to patients. It was reported that well-structured materials were particularly appreciated by patients as they were easy to understand and useful. However, respondents highlighted that they select and tailor the information provided for each patient to avoid overwhelming patients: *“But I don’t open my cupboard in every consultation and take out everything I have*,* because I think patients are very challenged at the beginning*,* sometimes even overwhelmed*,* and then they have to look at what is useful for me from these materials. What will help me? And if they have a mountain of information*,* they still have problems finding out for themselves what I need now and what I don’t need. I try to avoid that. I try to limit myself to what I think is helpful at the moment. If other aspects come up in the course of the process*,* then I usually have something up my sleeve.*” (A0101 pos. 42–47).

Only in one case was the project sustained in the long term. Beyond the duration of the project, some aspects, such as a decision guide or prompt cards were integrated into routine practice. The other projects and positions as a decision coach usually ended when funding stopped.

### Implementation process domain

We have summarized all aspects that affect or influence the implementation of the decision coaching intervention under this domain.

#### General training

Participants reported that the general multi-day training sessions were comprehensive, well-structured and effectively prepared them for practical application. However, practical experience was considered essential to further develop their skills in managing challenging interactions, such as dealing with uncertainty and emotional conversations with patients. Previous counselling experience or knowledge of specific diseases, such as breast cancer, was seen as beneficial by decision coaches.

#### Flexibility in implementation

The flexibility in the mode of delivery (face-to-face vs. online) was viewed positively. However, face-to-face sessions were perceived as more intensive and personal by the coaches: *“It’s not better. You should say it’s different. It’s just more personal in a face-to-face setting. The empathy and the interaction - there’s always something unique about it. I can see that very well online*,* yes. But sitting at the same table*,* makes it a different experience for me because I’m a very physical person. You can’t realise that now. I always use my hands; I use my whole face. You can see faces online*,* of course*,* but I’m more than that. I’m a whole person. And that’s how I perceive other people. I notice much more when someone is in the same room. And when tears flow*,* I feel able*,* even compelled*,* to comfort someone with a hug. A lot gets lost online.”* (A0105 pos. 18–25). However, the introduction of video consultations during the COVID-19 pandemic had the additional benefit that patients from outlying areas could participate and receive a decision coaching remotely.

The ability to adapt flexibly to the individual needs of patients was described as a significant advantage. Also noted as a beneficial aspect of decision coaching was the ability to involve family members or translators when needed.

Another positive mentioned factor was that the coaches are responsible for their own appointments and can schedule this flexibly in their own appointment system: *“It is good if you have your own calendar or*,* let’s say*,* if you can make appointments by your own. […] Or*,* for example*,* other employees can see that ‘Ah*,* Mrs A0404 has an appointment or a consultation that day‘. […] That’s certainly a big advantage.”* (A0104 pos. 62).

### Individuals domain

Within the individuals domain, we have summarized all aspects that concern both the decision coaching recipients (patients) and the deliverers (decision coaches), as well as the aspects related to the interaction between these two parties.

#### Further training, preparation and networking

Interviewees identified opportunities for further training as a crucial factor in their work as decision coaches. These opportunities were seen as a valuable complement to the introductory basic training that all coaches had received beforehand. In order to provide high quality decision coaching to patients, it is essential to keep up to date with the latest developments. It is therefore important that decision coaches have access to regular training opportunities: *“That’s the point where I say I would only be open to doing something like that again if I was really involved in further training.”* (A0106 pos. 319).

For the future, decision coaches also suggested that it would be beneficial to create opportunities for shadowing experienced coaches. This would allow new coaches to gain insight and experience within established structures. In addition, regular networking and exchange among decision coaches was seen as valuable to foster synergies and contribute to the further development of the coaching framework: *“Yes*,* and what I also think would be good if this were established is that there would be regular training. That you are really trained again*,* that it is refreshed and that there is also an exchange between decision coaches. That you can regularly exchange ideas about your work and get further training.”* (A0104 pos. 74).

#### Positive role understanding and identification with the role of a decision coach

A positive understanding of the role and a strong identification with the role were identified as beneficial. Coaches described a deep sense of personal fulfilment that they derived from their work, as well as a strong motivation to support patients in their decision making. *“I would do the coaching sessions again in a heartbeat. I just love the people and the contact with people and being able to support them*,* help them to help themselves and give them the knowledge to make a decision. I find that so valuable*,* it matches my way of thinking.”* (A0105 pos. 75).

#### Acceptance from patients

In terms of acceptance, all decision coaches reported that patients accepted the offer very well, regardless of the time of the consultation (just after diagnosis, already had the condition for some time, etc.). Only in a few cases, the patients refused the decision coaching offer or the decision coaches felt that the coaching did not go well: *“So the offer is for everyone*,* but it’s not necessarily helpful for everyone. Some people don’t want it. Usually*,* the acceptance is very good. Because in practice*,* of course*,* they don’t experience anyone sitting down with them for two hours.”* (A0103 pos. 48).

#### Benefits of decision coaching for patients

The interviewees described the benefits of decision coaching for patients as being that patients have more clarity about their own wishes and preferences regarding their illness and have a better understanding of the medical context and are therefore better prepared for discussions with their physicians: *“It is the case that when patients have undergone decision coaching and return to the physician*,* they find that they are well-informed and completely different from before.” (A0103*,* Pos. 67).*

### Inner setting domain

The inner setting domain includes social factors, such as local conditions or team-related aspects, as well as organizational factors and available resources.

#### Facilities and equipment

The decision coaches reported that a major barrier was the availability of rooms. In some cases, a room had to be shared with other staff members or no room was available at all. For each session, a free room within the clinic had to be located and booked. However, it could not be guaranteed that the available rooms were suitable in terms of atmosphere and equipment: *“I spent*,* I think*,* about three years in the on-call room for the residents. With a bed in the room. Terribly cramped. The atmosphere wasn’t very good for talking to patients either. Now I have a large office*,* but I have to share it with a colleague. That means I’m no longer in my room for consultations*,* where I can quickly go to the computer. I can print something out. Instead*,* I always have to go to a separate room for meetings.”* (A0101 pos. 58).

Another barrier may be the technical equipment of the rooms and the technology available to the coaches, which can complicate the implementation of coaching sessions: *“Just the general conditions […] so it’s small things*,* but they took away my own printer. We now have one in the whole corridor. That makes it more difficult*,* because if I want to give the patients something to take with them*,* something I want to look up quickly on the net or something else*,* then I can only do that by asking the patients to leave the room myself*,* by running to the printer and then back again*,* which stops the flow of information.”* (A0101 pos. 59).

#### Staffing and funding aspects

Other barriers that emerged from the interviews included staffing and their funding. The coaches emphasised the importance of having adequately trained staff. *“There is no one who can do this. Unless someone is trained.”* (A0105 pos. 74). This is necessary both to meet the demand for coaching and to ensure that the service can be provided during holidays or sick leave. It also needs to be clarified how the decision coaching can be refunded to ensure that appropriate conditions are in place.

#### Structural problems and limited room for decisions

Another barrier to decision coaching that emerged from the interviews is that in clinical practice, patients are often not given the space to make decisions, nor is it addressed that the decision could be made by the patients themselves. In some areas, after a diagnosis is made, patients are informed of their diagnosis over the phone by the treatment team, followed by an appointment for treatment or surgery. According to the coaches, in this process, patients are given little time or space to reflect on their diagnosis and treatment options and to decide about the next steps., *“But yes*,* it’s often the case that when we really talk about it*,* they suddenly say*,* ‘oh*,* this is the first time I’ve had time to think about my illness’*,* or sometimes only later.”* (A0102, Pos. 37). In addition, the coaches said that patients are often unaware that they are allowed and able to make decisions themselves. As a result, it can be challenging to identify the optimal timing for offering decision coaching and to integrate it into existing structures.

#### Acceptance and support from colleagues/physicians

Another barrier, but also a potential facilitator, is the acceptance and support of colleagues and physicians within the team. The interviewees emphasized the importance of informing all team members about the decision coaching and its potential benefits. Failure to do so can lead to misunderstanding and lack of support. This is particularly important when decision coaching is embedded in an interprofessional setting, where the identification of a decision conflict and the access to decision coaching depends on referrals from physicians. Their awareness of the benefits and procedures is essential for successful implementation.

#### Benefits of decision coaching for physicians

Interviewees described that both patients and physicians benefited from the decision coaching sessions. Patients were well prepared for the medical consultation after the coaching and were able to ask well-founded questions. This improved communication between physicians and patients: “*The physicians’ communication with the patients is actually made even easier by our presence.” (A0101*,* pos. 56)*. In addition, the interviewees felt that the coaching sessions saved time and reduced the physician’s workload, as questions could be clarified with the decision coaches beforehand: *“I think that a well-informed patient*,* who is really taken along at the beginning*,* has more resources for the marathon ahead of him in the long run and uses them less than a patient who is poorly informed and always needs new advice.”* (A0105 pos. 93).

#### Structural support from leadership

The decision coaches reported that the management level can be the biggest barrier but also the biggest supporter of the coaching programme: *“Support from the management. That it is wanted*,* that they see the sense in it*,* that you then of course have the support to be available for it or to get the time for it. That it is seen as so important. That you are supported.”* (A0104 pos. 64). It creates time and structural freedom for the coaches and makes the implementation of decision coaching possible in the first place. Coaches emphasise the value of having a free hand in designing the offer, which allows for innovative and flexible support. *“I had a completely free hand and we were in close contact. On a medical level*,* of course*,* with the chief physician*,* but also with the managing director*,* and that is a very*,* very important issue. And I think today’s CEOs come from the business side and not the medical side. They just have a different perspective.”* (A0101 pos. 75–76); *“So you need the support of the boss*,* otherwise it won’t work. Especially because it takes a lot of time.”* (A0103 pos. 77). Without the support of the management, a functioning and long-term implementation of decision coaching would not be possible: *“The chief physician has to be open to it*,* because if the chief physician is not open to it*,* then you can do what you want.”* (A0102 pos. 103).

### Outer setting domain

Within the *Outer Setting* domain, we identified key aspects related to the general understanding of the nursing role as well as health policy considerations based on the interview data.

#### General understanding of the nursing role

The interviewees also see the dominant societal understanding of the nursing role as a barrier to the implementation of decision coaching, as it is encountered in their work: *“Especially in the minds of physicians when we think about the medical field. I think many still have the impression that nursing is not capable of this kind of communication. But I think this applies to many areas of medicine*,* we have a system here that has always seen nursing as an assisting profession. One that assists or supports the physician*,* but has no expertise of its own. And I think politically a lot has to change in this regard.”* (A0101_pos. 63).

#### Funding and classification

Additionally, the issue of funding or classification, along with a clear role definition and clarification of the responsibilities of a decision coach, needs to be addressed: *“It’s more of a health policy issue*,* because it’s a bit more than what a nurse typically does*,* but less than a physician - something in between. It needs to be represented differently*,* not just in terms of appreciation*,* wanting it to happen in clinics or practices*,* but also financially.”* (A013, pos. 99). *“In my opinion we are not used or recognised as much as we could be. I think there’s still a lot of potential*,* but it’s not clear what the responsibilities are or what the role profiles are*,* and ultimately funding is linked to that.”* (A0101, pos. 90).

### Determinants of practice

**Fig. 1:**
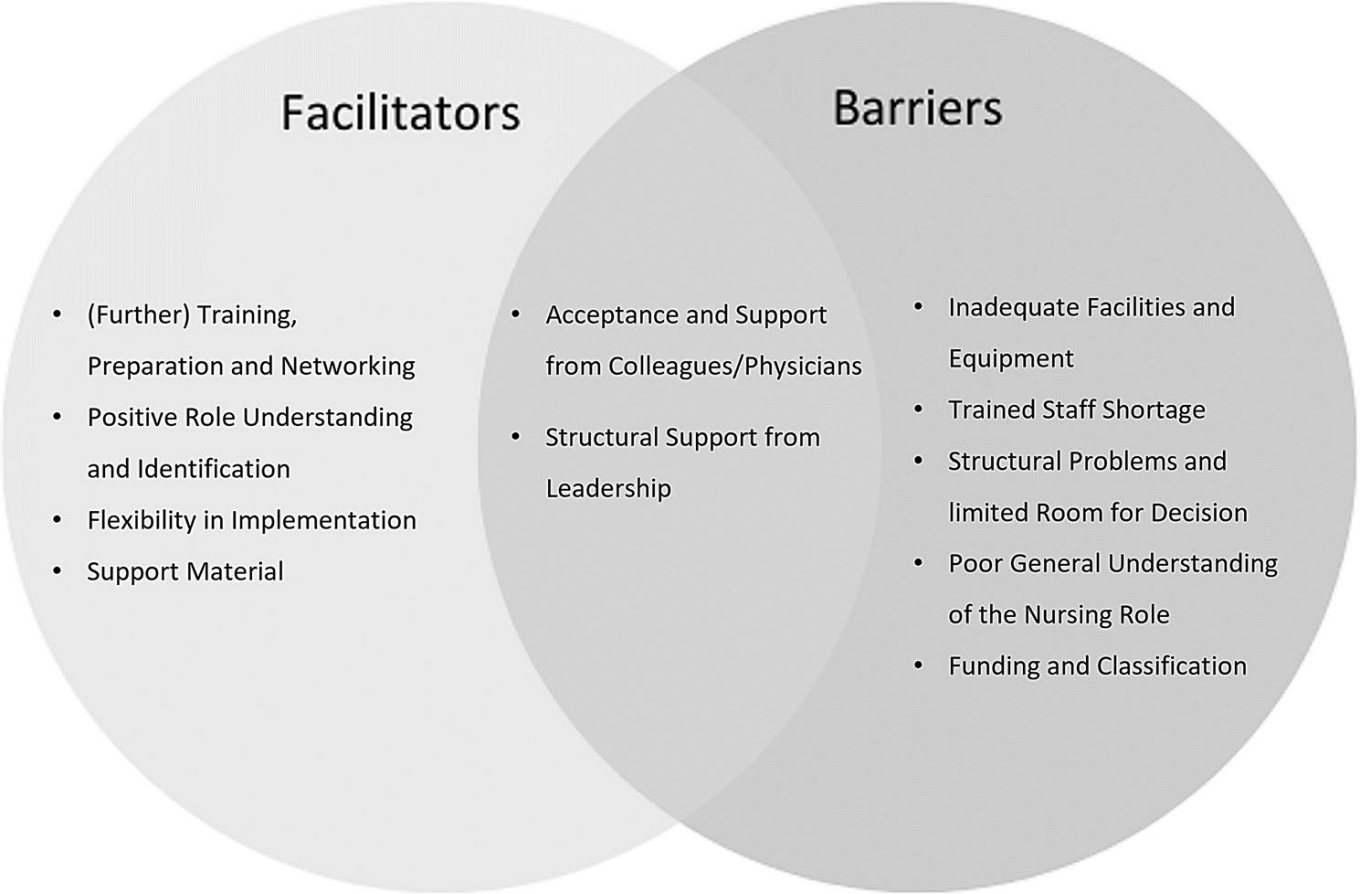
Identified facilitators and barriers of decision coaching

The identified practice determinants can be categorized as either potentially facilitating or potentially hindering. Additionally, some factors were found to have both facilitating and hindering effects (see Fig. 1). Positive outcomes for patients and physicians were also identified, resulting from participation in coaching, as reported by the decision coaches.

## Discussion

This study interviewed (former) decision coaches from Germany who were involved in decision coaching within model projects. It was possible to derive various practical determinants from the interviews. Of these, (further) training, preparation and networking, a positive understanding and identification of the role, flexibility in implementation and access to support materials can be classified as potentially promoting and inadequate facilities and equipment, limited human resources and funding, the current understanding of the role of nursing care and structural challenges, including limited room for decision-making as potentially hindering. Structural support from leadership and acceptance by colleagues and physicians emerged as dual factors that could act as either barriers or facilitators.

Our study highlights the importance of comprehensive and continuous training to ensure decision coaches feel confident in their role as decision coaches can implement decision coaching effectively. Studies have demonstrated that the personal commitment of the decision coaches plays a significant role in the successful implementation of decision coaching [25, 34, 35]. In addition, complex diseases such as cancer or chronic diseases require coaches to be constantly up to date. Rahn et al. evaluated a decision coaching program and reported that the coaches felt well prepared by the training [21, 22]. In their program theory, Zhao et al. also identified the commitment, skills and knowledge of decision coaches as critical factors for the successful implementation of decision coaching [20]. Comprehensive and ongoing training is essential to support these elements and maintain long-term effectiveness. Training multiple individuals also ensures continuity of service during illness or other absence. A cluster randomized controlled trial in the field of breast cancer in Germany also highlights the need for specific training. Not only for decision coaches, but for all team members. The authors suggest that this can increase the acceptance of coaches among physicians [25]. Alignment among the patient, decision coach, and physician toward the shared goal of patient involvement in decision-making is perceived as positive and supportive [20]. This is also reflected in our results, as acceptance by physicians, other colleagues and in particular by the leadership was identified as a key factor. Explaining the offer and emphasising the positive effects of the coaching sessions was mentioned as a way to increase acceptance and support. Leadership support is also suggested as a promising strategy to promote a shared vision of the decision coaching role across the team, which in turn can facilitate the implementation of decision coaching [20, 36]. In addition, leadership support could also have a positive impact on the facilities and resources available. Furthermore, it is well known that the support of the management level is essential for the integration of SDM in general [37]. For the successful implementation of SDM and decision support interventions such as decision coaching, it is crucial that these are firmly embedded in the structures and are part of the care provided.

From the perspective of decision coaches, a key challenge is that patients often do not realise that they can make a decision, as they are rarely given adequate time or space to make informed decision. According to the coaches, the short time between diagnosis and the start of treatment frequently means that patients only had the opportunity to reflect on their diagnosis and treatment options for the first time during the coaching sessions. Studies show that patients often feel inadequately informed and believe they need more information than they have received [3, 4] and many are unaware that they have a choice regarding their treatment options [38]. Time constraints in clinical settings, as well as the urgency of some medical conditions, often limit the time available for consultation and decision-making [39, 40]. It has also been described that the time available for the decision-making process is often insufficient for patients to participate adequately [41, 42]. It is crucial for patients, especially those who have received bad news, to have sufficient time for decision-making in order to actively participate in the shared decision-making process [41–43]. However, the amount of time needed varies and must be tailored to the individual patient [42]. It is important to overcome these time barriers and the imbalance in the communication of information and the need for information. Decision coaching can act as a link in overcoming these barriers by encouraging and preparing patients to actively participate in the decision-making process. Actively inviting patients to participate can be key factor in encouraging their engagement in decision support [8, 20]. In addition, concerns should be addressed early in the process [42] and it should also be clarified at the initial stage where the patient currently stands in the decision-making process (e.g., whether they have already started considering the options) [8]. Additionally, the available time for decision-making and the timeframe within which a decision should be made should be discussed with the patient and healthcare team at the start [42]. In future studies, it would also be important to find out at what point in the decision-making process decision coaching is most useful and efficient for patients and also for the healthcare team.

Another important aspect was the ability to tailor coaching sessions to the individual needs and preferences of patients, allowing for a flexible and patient-centered approach. This adaptability helped foster a trusting and comfortable atmosphere. Several studies also highlight trust and a needs-oriented approach as key factors in the success of decision coaching [20, 34, 35].

This also aligns with the use of additional support tools, such as decision aids or evidence-based health information. The use of those tools was described as positive by the coaches if their use was tailored to the decisional needs of the patients. The combining of decision coaching with evidence-based health information could improve patients’ knowledge and active participation in decision-making [11, 19]. However, it is important to consider that the studies reporting these results all had small sample sizes. The use of decision aids may, e.g., lead to an increase in patient knowledge, a better understanding of risks and a reduction in decisional conflict [10]. However, the effectiveness of combining decision aids with decision coaching needs to be investigated in the future.

### Strength and limitations

The chosen method of data collection can be seen as a strength of our study. The individual interviews provide a deep insight into the personal experiences of the decision coaches. At the same time, it is possible to respond individually to what is said and thus to address issues that may not have been considered in advance in the interview guidelines. In addition, the decision coaches are able to express their experiences in their own words, which allows for an authentic and unfiltered account. In addition, the use of the CFIR 2.0 framework can be seen as a strength. It is a well-recognised implementation framework, which ensured a structured and comprehensive approach to analyse the determinants of decision coaching practice. Furthermore, this analysis also enables the targeted selection of implementation strategies and the further development of decision coaching interventions.

However, our study has some limitations. The data analysis was carried out by only one researcher (LS). To minimise this limitation, a discussion of single elements like the coding guide was carried out with other experienced qualitative researchers of the research community. Another limitation is the small sample size of seven participants, which may not capture the range of perspectives or relevant topics within the target group. The sample size was constrained by the specificity and limited accessibility of the target population as there are only very few decision coaches in Germany. Additionally, only female participants were included, limiting the ability to consider gender-diverse perspectives. Nevertheless, the sample did include coaches from various areas of care, contributing to a broader representation within these constraints.

Data saturation was taken into account during data collection. Given the very limited number of decision coaches currently available in Germany, our study population was necessarily small. Despite this constraint, the interviews revealed consistency. Similar themes recurred across participants, and their statements often overlapped or confirmed each other. This repetition of central ideas and the absence of substantially new information in later interviews suggested that thematic saturation had been achieved, even within the restricted sample size.

In addition, this study only shows the perspective and experience of the decision coaches. The aspects reported regarding the acceptance of patients and other interest holders are based on the subjective perceptions of the decision coaches. For a comprehensive picture, other perspectives such those from the group of patients, physicians and leadership figures should also be involved. Experience from the leadership level, for example, would be essential for topics such as funding or why the service could not be incorporated into the regular care structures. Most of the decision coaches in this study were no longer actively involved in the decision coaching projects at the time of the interviews. Their roles were tied to temporary project funding, so their termination was due to the end of that funding. Since the decision coaches had limited involvement in organizational decision-making processes, they could not provide further insides into why the project was not continued. This limitation leaves unanswered questions about the structural, financial, and institutional factors that contributed to the termination of these projects. Future research with larger and more diverse samples is needed to confirm and extend the existing findings.

## Conclusion

Decision coaches perceive themselves as key facilitators in empowering patients to navigate through complex medical decisions, ensuring that choices are consistent with their values and preferences. However, the effectiveness and successful integration of decision coaching into healthcare settings is highly dependent on several influencing factors. These factors need to be carefully addressed to maximise the potential of decision coaching. It is important to note that this study involved a small target group. Future research should include larger sample sizes and different perspectives, particularly those of patients, healthcare leaders and physicians, to gain a more comprehensive understanding of the determinants of practice. Such studies would provide a broader and more nuanced basis for implementing and optimising decision coaching in practice.

## Electronic Supplementary Material

Below is the link to the electronic supplementary material.

Supplementary Material 1

Supplementary Material 2

Supplementary Material 3

## Data Availability

Full access to the raw data is available upon reasonable request.

## Abbreviations

CFIR 2.0: Consolidated Framework for Implementation Research
ODSF: Ottawa Decision Support Framework
SDM: Shared Decision Making
