# Supplementary material for: Determinants of practice for decision coaching in Germany: a qualitative exploration of decision coaches’ perspectives
Source: BMC Health Serv Res. 2025 Dec 1;25:1560. doi: 10.1186/s12913-025-13752-z (PMC12670830; doi:10.1186/s12913-025-13752-z)
Supplement: Supplementary file 1 — Supplementary Material 1 [file 12913_2025_13752_MOESM1_ESM.pdf]

Supplementary Material 1: COREQ (COnsolidated criteria for REporting Qualitative research) Checklist

To pic	Item No.	Guide Questions/Description	Reported on Page No.
**Domain 1: Research team and reflexivity**			
Personal characteristics			
Interviewer/facilitator	1	Which author/s conducted the interview or focus group?	7
Credentials	2	What were the researcher's credentials? E.g. PhD, MD	1
Occupation	3	What was their occupation at the time of the study?	1
Gender	4	Was the researcher male or female?	1
Experience and training	5	What experience or training did the researcher have?	24
Relationship with participants			
Relationship established	6	Was a relationship established prior to study commencement?	n.a.
Participant knowledge of the interviewer	7	What did the participants know about the researcher? e.g. personal goals, reasons for doing the research	n.a.
Interviewer characteristics	8	What characteristics were reported about the inter viewer/facilitator? e.g. Bias, assumptions, reasons and interests in the research topic	24
**Domain 2: Study design**			
Theoretical framework			
Methodological orientation and Theory	9	What methodological orientation was stated to underpin the study? e.g. grounded theory, discourse analysis, ethnography, phenomenology, content analysis	6 ff
Participant selection			
Sampling	10	How were participants selected? e.g. purposive, convenience, consecutive, snowball	7
Method of approach	11	How were participants approached? e.g. face-to-face, telephone, mail, email	7
Sample size	12	How many participants were in the study?	8
Non-participation	13	How many people refused to participate or dropped out? Reasons?	8
Setting			
Setting of data collection	14	Where was the data collected? e.g. home, clinic, workplace	8
Presence of non-participants	15	Was anyone else present besides the participants and researchers?	8
Description of sample	16	What are the important characteristics of the sample? e.g. demographic data, date	9
Data collection			
Interview guide	17	Were questions, prompts, guides provided by the authors? Was it pilot tested?	8
Repeat interviews	18	Were repeat interviews carried out? If yes, how many?	n.a.
Audio/visual recording	19	Did the research use audio or visual recording to collect the data?	7
Field notes	20	Were field notes made during and/or after the interview or focus group?	7
Duration	21	What was the duration of the interviews or focus group?	8

Data saturation	22	Was data saturation discussed?	18 ff
Transcripts returned	23	Were transcripts returned to participants for comment and/or correction?	7

**Domain 3: analysis and findings**			
Data analysis			
Number of data coders	24	How many data coders coded the data?	8
Description of the coding tree	25	Did authors provide a description of the coding tree?	8
Derivation of themes	26	Were themes identified in advance or derived from the data?	8
Software	27	What software, if applicable, was used to manage the data?	8
Participant checking	28	Did participants provide feedback on the findings?	n.a.
Reporting			
Quotations presented	29	Were participant quotations presented to illustrate the themes/findings?<br>Was each quotation identified? e.g. participant number	9 ff
Data and findings consistent	30	Was there consistency between the data presented and the findings?	18 ff
Clarity of major themes	31	Were major themes clearly presented in the findings?	9 ff
Clarity of minor themes	32	Is there a description of diverse cases or discussion of minor themes?	9 ff
