# Supplementary material for: Determinants of practice for decision coaching in Germany: a qualitative exploration of decision coaches’ perspectives
Source: BMC Health Serv Res. 2025 Dec 1;25:1560. doi: 10.1186/s12913-025-13752-z (PMC12670830; doi:10.1186/s12913-025-13752-z)
Supplement: Supplementary file 2 — Supplementary Material 2 [file 12913_2025_13752_MOESM2_ESM.pdf]

Table 1: Main Categories and Descriptions

**Main Category**<br>*Subcategory*		
**General Information about Decision Coaching**	This code is assigned when general information on the task of decision coaching is given.	
Time off for Work	This code is assigned when respondents report whether they were given time off from their job to act as a decision coach.	"So, I was actually given time off. Not true. The time I spent coaching, etc., I obviously didn't do in my free time. So that's a wrong term. No, of course I was given time off." (Interview_A0105, pos. 64)
Self-perception of Role	This code is assigned when participants describe what the role of a decision coach means to them personally.	"Yes. I have to say honestly, I would immediately conduct the coaching again. I just love people and the contact with them, supporting people towards self-help, giving help towards self-help and conveying knowledge so they can make a decision. I find that so valuable; it fits my thinking." (Interview_A0105, pos. 75)
Task Description	This code is assigned when respondents describe their tasks/role as decision coach from their perspective.	"And I managed the study alone. So, I recruited the patients, conducted the coaching, the follow-up—everything by myself, and that was great." (Interview_A0105, pos. 2)
Preparation for Decision Coaching	This code is assigned when respondents explain how they were prepared for the role of decision coach, e.g., through training.	"The training took place in Hamburg. I found it very comprehensive, very well structured, and we also received the appropriate materials we worked with during the study. I still have them in my cabinet. From time to time, I look at them again, especially since cancer patients sometimes struggle more with considering whether or not to agree to a radiotherapy. I always emphasize that it's old data, but it still helps make the point of treatment clearer. So, I still use it." (Interview_A0101, pos. 8-10)
Duration of Activity	This code is assigned when respondents describe how long they worked as decision coaches.	"Yes, I've been doing this for a long time, a very long time, but in this intensity, for two years." (Interview_A0102, pos. 76-77)

**Framework Conditions**	This code is assigned when respondents describe the framework conditions of decision coaching in more detail.	
Funding	This code is assigned when aspects of funding are addressed.	“However, I believe that cost coverage is often complicated in such cases, for example when patients are not physically present. It is difficult to have phone consultations and similar interactions refunded appropriately.” (Interview_A0103, pos. 91)
Human Resources	This code is assigned when aspects of human resources are addressed.	"There’s just very little specialized staff, and even fewer who are trained. That was already a problem during the study. It’s just not possible to recruit more people or pull staff from other areas." (Interview_A0105, Pos. 101)
Integration into Hospital Structures	This code is assigned when the integration of decision coaching into the hospital or practice routines is described.	"This is part of the day clinic. We’re an outpatient clinic, a day clinic, and study center. Patients come to us as part of the day clinic, and the decision coaching is firmly anchored with two billable appointments." (Interview_A0103, pos. 5)
Facilities	This code is assigned when the rooms where coaching took place are described.	"Depending on the time of my training sessions, rooms were already booked. Then I looked around and always managed to find a space for us." (Interview_A0105, pos. 31)
Theoretical planned Process	This code is assigned when respondents describe the planned process of a coaching session.	"We plan two appointments: first for prognosis and diagnosis, and second for treatment. The first takes about an hour. The second is shorter—around 20 minutes—when they already know what they want." (Interview_A0103, pos. 27)
Implementation Context	This code is assigned when it is explained in which context the coaching took place (e.g., study).	"I should mention that diagnoses are given over the phone. That’s a disaster. If they’ve been biopsied, our senior doctors often already know if it's malignant or not. But they must wait a week for a phone call." (Interview_A0102, pos. 29)
Mode of Delivery of the Decision Coaching	This code is assigned when it is stated whether coaching was offered on-site or online.	"All on-site. One-on-one conversations, or with relatives—then maybe six or eight eyes. Even during

		the COVID period, we held everything in person under proper hygiene rules." (Interview_A0101, pos. 11-12)
Decision Coaching Frequency	This code is assigned when coaching frequency is addressed.	"There is a fixed structure. Some appointments are planned, but it depends on the patients' needs. They call, come by, or email me. Sometimes we handle things over the phone or plan another appointment." (Interview_A0101, pos. 30-31)
Duration of Coaching	This code is assigned when respondents describe the typical duration of a coaching session.	"They usually lasted about an hour to one and a half hours." (Interview_A0107, pos. 7)
**Support Needs of Decision Coaches**	This code is used when the decision coaches report in which area they would like to receive support.	
Support Tools	This code is used when interviewees report that they would like to have more support tools (e.g., patient decision aids) to assist them in their work.	"I: So, what I'm also hearing is that if you had statistics, you would actually use them."<br>"A0202: Yes, exactly. Then I would use them."<br>(Interview_A0102, Pos. 88–89)
Networking	This code is used when coaches express a desire for regular peer exchange with other decision coaches.	"There are definitely some areas that are interesting, but I'm not sure. It probably needs to be made more public, and there should be more networks or collaborations among decision coaches." (Interview_A0103, Pos. 97–99)
Further Training	This code is applied when it is reported that decision coaches wish to receive regular further training	"When decision coaching is implemented, I believe it is essential to ensure that coaches receive regular updates and stay informed." (Interview_A0106, Pos. 319)
**Utilization**	This code is assigned when respondents describe who and how decision coaching was utilized.	
Target Group	This code is assigned when respondents describe the characteristics and related needs of people who made use of the decision coaching.	"Especially after tumor boards, when it's about decision-making or receiving information, women are often so nervous that they like to bring a partner. Or with very elderly patients, they bring their children. Or if they are non-native speakers, they of course need someone to translate." (Interview_A0101, pos. 37-38)

Timing of Coaching	This code is assigned when respondents describe when a decision coaching was scheduled.	"Rarely, but rather when it's not a high-risk woman, and it seems like surgery and maybe radiotherapy will be enough. Then it may be that the women come to me after the tumor board." (Interview_A0101, pos. 25)
Emotional Access to Patients	This code is assigned when the accessibility of patients is described with a focus on emotional access.	"You're right. The content and nature of the conversations vary from patient to patient. Rarely, I feel like I'm talking to a wall. Sometimes I can't find access, which may be due to extreme fear, or simply no chemistry." (Interview_A0101, pos. 40)
Decision Situation	This code is assigned when respondents describe the decision-making situations in their coaching.	"For those who had just received their results, they usually said 'I'm uncertain' or 'Everything is an option for me.' But there were also women where it depended on their family history." (Inteview_A0104, pos. 32)
Decision Coaching Topics	This code is assigned when respondents elaborate on the specific topics addressed during coaching.	"If the ovaries are removed. Or the question of intensive monitoring in the tumor risk consultation or risk-reducing breast surgery. And if choosing surgery, which type. Those were the topics." (Interview_A0106, pos. 261)
Use of Decision Support Tools	This code is assigned when respondents describe the use and benefit of decision support tools (e.g., decision aids) in their coaching.	"We developed a lot of our own handouts. For each side effect, there are many aids—those are my tools." (Interview_A0102, pos. 9)
**Benefits from Decision Coaching**	This code is assigned when respondents report benefits that arise from decision coaching from their perspective.	
Benefits for Physicians	This code is assigned when benefits of decision coaching for physicians are described.	"It also relieves the physicians. I could imagine being a go-to person outside coaching sessions for interim questions—nurses are often more accessible than physicians." (Interview_A0104, pos. 46)
Benefits for Patients	This code is assigned when benefits of decision coaching for patients are described.	"Women often already have the information in medical consultation. It's not all new—they understand and apply it better." (Interview_A0101, pos. 56)

**Acceptance**	This code is assigned when the acceptance of the various groups of people involved in decision coaching is described.	
Patients	This code is assigned when the acceptance of patients is described.	"None reacted negatively. Especially younger women or newly diagnosed ones were very positive and had lots of questions we could clarify." (Interview_A0107, pos. 157)
Physicians	This code is assigned when the acceptance of physicians is described.	"Acceptance was high from the start. At first, physicians just let me do my thing. I had full freedom and even defined my own job description." (Interview_A0101, pos. 53)
Management	This code is assigned when the acceptance from hospital/practice leadership is described	"But ultimately, yes. From management—no obstacles. It was accepted and desired that the study ran." (Interview_A0105, pos. 72)
Colleagues	This code is assigned when the acceptance from colleagues is described.	"Well, as I said, the acceptance from colleagues. They don't see what you're doing—just that you're gone, absent." (Interview_A0105, pos. 71)
**Sustainability and Maintenance**	This code is assigned when respondents provide information on whether and how their offer has been consolidated.	
Use beyond the Study	This code is used when it is reported that partial content or tools are used beyond the end of the study.	"I do give them information about how the therapies work, because I also help them with that—I actually provide quite a bit of counselling around all the treatment options. But the patients themselves don't get any statistics anymore." (Interview_A0102, pos. 3)
Continuation	This code is assigned if the offer has been made permanent. The code is also assigned if the respondents give reasons for the continuation.	"And it will continue beyond the study?<br>A0103: Yes, exactly." (Interview_A0103, pos. 20-21)
Discontinuation	This code is assigned if the offer was not made permanent. The code is also assigned if the respondents give reasons against the continuation.	"Mhm, so since the end of the study I've stopped doing that because, as I said, it hasn't been implemented yet." (Interview_A0101, pos. 2)
