# Supplementary material for: Determinants of practice for decision coaching in Germany: a qualitative exploration of decision coaches’ perspectives
Source: BMC Health Serv Res. 2025 Dec 1;25:1560. doi: 10.1186/s12913-025-13752-z (PMC12670830; doi:10.1186/s12913-025-13752-z)
Supplement: Supplementary file 3 — Supplementary Material 3 [file 12913_2025_13752_MOESM3_ESM.pdf]

Universität Bremen

TARGET
Vernetzte Versorgung bei Krebs

Interview Guide

**Interview**	
**Introduction**<br>The interviews are conducted as part of a doctoral research project aiming to develop and evaluate decision support tools for individuals seeking advice in oncology. They serve to explore the decision coaching support service in more detail and to identify potential barriers and facilitators to its implementation.	
**General Information about Decision Coaching**	**5 min**
You have taken on the role of a decision coach in the past or may still be doing so. Could you describe what that involves and what your work as a decision coach looks or looked like?	
How would you describe your role as a decision coach?	
In what context did you provide decision coaching?	
How were you prepared for this role?<br>Did you attend any specific training?<br>Did you feel sufficiently prepared for the coaching sessions?	
How long have you been (or were you) active as a decision coach?	
How did you conduct the coaching sessions (in person, by phone, online)?	
How much time was allocated for each session, and approximately how long did they last?	
**Information on the Use of Decision Coaching**	**10 min**
What kind of individuals seek you out or make use of the coaching service?<br>To what extent do people come alone or accompanied by family or friends?	
What are the typical reasons for seeking advice? What kinds of topics or decision-making situations are brought to you?	
What are the most common decision-making situations you encounter in your daily coaching practice?	
What are the most frequent decision-making needs in your coaching sessions?	
To what extent are tools (e.g., decision aids) used in your coaching?<br>If so, which tools do you use?<br>In what way has the use of these tools had a positive or negative impact on the coaching process?	
**Framework Conditions**	**5 min**
What did the theoretically planned course of a coaching session look like?<br>To what extent did your actual practice deviate from this plan?<br>If deviations occurred, what were the reasons?	

Universität Bremen

TARGET
Vernetzte Versorgung bei Krebs

How did individuals seeking advice become aware of the decision coaching service?<br>From your perspective, were there any difficulties or areas for improvement in this regard?<br>How was the decision coaching service integrated into the structural workflows of the hospital, practice, or other setting?	
**Acceptance**	**5 min**
How would you describe the acceptance of decision coaching by:<br>a) Patients<br>b) Physicians<br>c) Management<br>d) Other colleagues	
How was the decision coaching service received by patients, from your perspective?<br>If acceptance was limited, what do you think were the reasons?<br>Where do you see potential for improving acceptance?	
**Barriers and Opportunities**	**5 min**
Beyond what has already been mentioned, were there any additional barriers that made it difficult for individuals to access or for you to deliver the coaching?	
What general barriers or challenges do you see for the implementation of decision coaching?	
In your view, what opportunities or facilitating factors exist for successful decision coaching?	
**Sustainability**	
If the service was initially offered as part of a project, to what extent has it been sustained beyond the project phase?<br>What factors contributed to its continued implementation?<br>What factors made it difficult to sustain the service?	
What influence do other colleagues and physicians have on the decision coaching service?	
Thank you very much for your participation and feedback. Is there anything else you would like to share with us that you feel hasn't been covered yet?	**5 min**
